# No abatement of steroid injections for tennis elbow in Australian General Practice: A 15-year observational study with random general practitioner sampling

**DOI:** 10.1371/journal.pone.0181631

**Published:** 2017-07-20

**Authors:** Bill Vicenzino, Helena Britt, Allan J. Pollack, Michelle Hall, Kim L. Bennell, David J. Hunter

**Affiliations:** 1 School of Health and Rehabilitation Sciences: Physiotherapy, University of Queensland, Queensland, Australia; 2 Family Medicine Research Centre, University of Sydney, Sydney, New South Wales, Australia; 3 Centre for Health, Exercise and Sports Medicine, Department of Physiotherapy, University of Melbourne, Melbourne, Australia; 4 Institute of Bone and Joint Research, Kolling Institute, and University of Sydney, Sydney, New South Wales; University of South Australia, AUSTRALIA

## Abstract

**Objective:**

Evaluate general practitioner (GP) management of tennis elbow (TE) in Australia.

**Methods:**

Data about the management of TE by GPs from 2000 to 2015 were extracted from the Bettering the Evaluation of Care of Health program database. Patient and GP characteristics and encounter management data were classified by the International Classification of Primary Care, version 2, and reported using descriptive statistics with point estimates and 95% confidence intervals.

**Results:**

TE was managed by GPs 242,000 times per year on average. Patients were mainly female (52.3%), aged between 35 and 64 years (mean: 49.3 yrs), had higher relative risks of concomitant disorders (e.g. carpal tunnel syndrome and other tendonitis) and their TE was 10 times more likely to be work related than problems managed for patients who did not have TE. Use of diagnostic tests was low, implying a clinical examination based diagnosis of TE. Management was by procedural treatments (36 per 100 TE problems), advice, education or counselling (25 per 100), and referral to other health care providers (14 per 100, mainly to physiotherapy). The rate of local injection did not change over the 15 years and was performed at similar rates as physiotherapy referral.

**Conclusion:**

The high risk of comorbidities and work relatedness and no abatement in the reasonably high rate of local injections (which is contrary to the evidence from clinical trials) provides support for the development and dissemination of TE clinical guidelines for GPs.

## Introduction

Tennis elbow is a common term describing an overuse condition that results in pain over the lateral elbow provoked by activities involving gripping and manipulating objects with the hand. It usually presents in mid-life in both male and females equally. Tennis elbow is frequently a consequence of participation in activities that involve unaccustomed (i.e. inadequately prepared for) repetitive manual tasks that require gripping an implement, often in awkward wrist positions, such as in meat processing, gardening, building, and tennis[1]. It is relatively common, although rates depend upon the specific population. For example, point prevalence rates range from 1.1 to 1.3% in the general community[2] but have been reported as being 5% in males and 11% in females in meat processing factories[3] and 14% in members of a private tennis club[4].

Management of tennis elbow can vary from asking the patient to do no active treatment by adopting a ‘wait and see’ approach, to many different forms of physical therapies (including exercise), injections, or to surgery in a minority of recalcitrant cases[5]. Evidence indicates that while injections might help in the short term, in the longer term (3–12 months) they tend to lead to delayed healing and higher recurrence rates than a ‘wait and see’ approach[6]. A recent network comparison analysis showed that physical therapies such as exercise, laser, acupuncture and manual therapy, as well as multimodal (combination of) physical therapies, have a beneficial effect over control (e.g. wait and see) or placebo comparators[7].

Managing a condition with such a range of potential treatment options is likely challenging for practitioners and their patients. The aim of this study was to evaluate tennis elbow in Australian general medical practice in terms of its management frequency, treatments delivered, referrals and associated general medical practice demographics. Studying these practice patterns will assist in the development of guidelines that aid clinicians with their decision making when managing tennis elbow.

## Materials and methods

We analysed data from the Bettering the Evaluation and Care of Health (BEACH) program, the methods of which have been described in detail elsewhere[8, 9] In brief, each year approximately 1000 GPs across Australia are randomly sampled from Australian government GP Medicare (Australia’s universal health scheme) claims records. Approximately 80% of those who agree to participate, record details of 100 consecutive encounters with consenting patients, including up to four problems dealt with (‘managed’) at each encounter. Each problem is linked by the recording GP to any resulting clinical actions such as medications, clinical and procedural treatments, tests and referrals. GPs record information in free text on structured paper forms. Completed forms are returned to the research team. Problems managed and all non-pharmacological management actions are coded and data entered by trained secondary clinical coders according to the International Classification of Primary Care, Version 2 (ICPC-2)[10], but are coded more specifically using the Australian GP interface terminology known as ICPC-2 Plus [11]. The GP and encounter samples from BEACH each year have repeatedly been shown to be representative of GPs and their patient encounters across the country. It is not meant to represent the population but rather represent GP activity[8, 9]. The BEACH program is approved by the Human Research Ethics Committee of the University of Sydney (reference 11428).

In this study we analysed all encounters between April 2000 and March 2015 inclusive, at which TE was managed (as a new or previously diagnosed problem). New problems were defined as either a first presentation of TE or the first presentation of a recurrence of previously resolved TE, whereas old problems were those previously diagnosed for which continuing care was being given. TE was defined as “L93” in ICPC-2 and includes the Plus terms [11] “Epicondylitis”, “Epicondylitis;elbow”, “Epicondylitis;lateral”, “Tendonitis;elbow”, “Tennis elbow” and “Tenosynovitis;elbow”, as coded by secondary clinical coders in ICPC-2 PLUS, an Australian general medical practice interface terminology. Medications (up to 4 per TE problem) were coded using the Coding Atlas of Pharmaceutical Substances (CAPS)[12], which is classified according to the Anatomical Therapeutic Chemical classification (World Health Organization)[13].

Statistical procedures were performed in SAS 9.3 © (SAS Institute Inc., Cary, NC), adjusted for the cluster survey design and for individual GP activity (as measured by total claims in previous 12 months from Medicare). When comparing any two comparable groups, differences were considered statistically significant if *P*<0.05, which includes the criterion of ‘non-overlapping 95% confidence intervals (CIs)’ (*P*<0.006) [14]. Use of this criterion is a conservative approach, which decreases the risk of Type I error, but increases the risk of Type II error.

## Results

### Frequency of TE managed

TE was managed at 3181 (0.22%; 95% CI: 0.21–0.22) of 1,471,600 recorded encounters. This extrapolates to an average of approximately 242,000 (95% CI: 233,000–251,000) encounters per year nationally, at which TE was managed. Between the years 2000-01 and 2014-15, there was a small linear decrease in the proportion of total encounters involving management of TE (odds ratio 0.98 (95% CI: 0.97–0.99), *P*<0.0001).

### Description of patient and GP characteristics

Patients for whom TE was managed were more often: female; aged 35–64 years; of English-speaking background; and non-Indigenous (Table 1). The characteristic-specific management rates showed that the likelihood of TE being managed was higher at encounters with patients who were: male (0.25%, versus female 0.19%); aged 45–54 years; of non-English speaking background; and non-Indigenous (Table 1). There was no difference in the age and sex distributions of patients at encounters where TE was a new problem (n = 1609 encounters) and where it was an old problem (n = 1573 encounters)(data not tabled).

**Table 1:** Sex, age and other group distributions and group-specific likelihoods of patients presenting with tennis elbow (TE) problem at encounter (April 2000 –March 2015).

Sex*	Number of encounters at which TE was managed	% sex distribution (95% CI)	Sex-specific likelihood (%) of TE (95% CI)
**Male**	**1,505**	**47.7 (45.8–49.5)**	**0.25 (0.24–0.27)**
**Female**	**1,652**	**52.3 (50.5–54.2)**	**0.19 (0.18–0.20)**
**All known sex**	**3,157**	**100.0**	** **
Age group* **(years)**	**Number of encounters at which TE was managed**	**% age distribution (95% CI)**	**Age-specific likelihood (%) of TE (95% CI)^**
**< 1 year**	**1**	**0.0 (0.0–0.1)**	**0.00 (0.00–0.01)**
**1–4 years**	**0**	**. (.–.)**	**0.00 (0.00–0.00)**
**5–14 years**	**13**	**0.4 (0.2–0.6)**	**0.02 (0.01–0.03)**
**15–24 years**	**65**	**2.1 (1.5–2.6)**	**0.05 (0.04–0.06)**
**25–34 years**	**199**	**6.3 (5.4–7.2)**	**0.12 (0.10–0.14)**
**35–44 years**	**827**	**26.2 (24.6–27.8)**	**0.46 (0.43–0.50)**
**45–54 years^**	**1,171**	**37.1 (35.3–38.8)**	**0.59 (0.55–0.62)**
**55–64 years**	**616**	**19.5 (18.1–20.9)**	**0.31 (0.28–0.33)**
**65–74 years**	**181**	**5.7 (4.9–6.6)**	**0.10 (0.08–0.11)**
**75+ years**	**86**	**2.7 (2.1–3.3)**	**0.04 (0.03–0.05)**
**All known age**	**3,159**	**100.0**	** **
Non-English speaking background (NESB) status*	**Number of encounters at which TE was managed**	**% NESB status distribution (95% CI)**	**NESB-specific likelihood (%) of TE (95% CI)**
**Non-English speaking background**	**303**	**10.6 (9.2–11.9)**	**0.27 (0.23–0.30)**
**English speaking background**	**2565**	**89.4 (88.1–90.8)**	**0.21 (0.20–0.22)**
Indigenous status*	**Number of encounters at which TE was managed**	**% Indigenous status distribution (95% CI)**	**Indigenous status-specific likelihood (%) of TE (95% CI)**
**Indigenous**	**32**	**1.2 (0.8–1.6)**	**0.15 (0.10–0.20)**
**Non-Indigenous**	**2606**	**98.8 (98.4–99.2)**	**0.21 (0.21–0.22)**
Australian Statistical Geography Standard (ASGS)*	**Number of encounters at which TE was managed**	**% ASGS distribution (95% CI)**	**ASGS-specific likelihood (%) of TE managed at encounter (95% CI)**
**Major Cities**	**2186**	**70.7 (68.8–72.5)**	**0.22 (0.21–0.23)**
**Inner Regional**	**592**	**19.1 (17.5–20.7)**	**0.22 (0.20–0.24)**
**Outer Regional**	**263**	**8.5 (7.4–9.6)**	**0.20 (0.18–0.23)**
**Remote**	**37**	**1.2 (0.8–1.6)**	**0.23 (0.16–0.30)**
**Very Remote**	**16**	**0.5 (0.2–0.8)**	**0.20 (0.10–0.30)**

*Missing: Of 1,471,600 encounters in total, the numbers of missing for each group were as follows: sex 13,262, age 12,385, Non-English speaking background 142,171, Indigenous status 236,818, and ASGS 39,140. Of 3181 encounters where TE was managed, the numbers of missing for each group were as follows: sex 24, age 22, NESB 313; Indigenous status 543; and ASGS 87.

^Example: As an example, of 198,900 encounters with patients aged 45–54 years, the likelihood that TE was managed at any one of these encounters was 0.59% (95% CI: 0.55–0.62); i.e. TE was managed at approximately 6 per 1000 encounters with patients aged 45–54 years.

Of the 14,716 GP participants, 2654 (18.0%, 95%CI: 17.4–18.7) managed TE at least once in their 100 recorded encounters. Female GPs (15.1%, 95%CI: 14.2–16.1) were less likely than male GPs (19.8%, 95%CI: 19.0–20.6) to manage TE at any of their 100 encounters (Table 2).

**Table 2:** GP participant sex and age distributions and group-specific likelihoods for tennis elbow (TE) problem at encounter (April 2000 –March 2015).

**Sex**	Number of GPs who managed at least one TE problem at any of the 100 encounters	% sex distribution of these GPs	^GP sex-specific likelihood (%) of managing TE at any of the 100 encounters (95% CI)^
**Male^**	1,824	68.7	19.8 (19.0–20.6)
**Female**	830	31.3	15.1 (14.2–16.1)
**All known sex**	**2,654**	**100.0**	** **
Age group (years)*	**Number of GPs who managed at least one TE problem at any of the 100 encounters**	**% age distribution of these GPs**	**GP age-specific likelihood (%) of managing TE at any of the 100 encounters (95% CI)^**
**<35 years**	158	6.0	16.2 (13.9–18.5)
**35–44 years**	612	23.2	19.3 (17.9–20.7)
**45–54 years**	945	35.8	18.9 (17.8–20.0)
**55+ years**	922	35.0	16.8 (15.8–17.8)
**All known age**	**2,637**	**100.0**	** **

*Missing: Of 14,716 GP participants in total, GP age was missing for 90. Of 2,654 GPs who managed at least one TE problem at any of the 100 encounters, age was missing for 17.

^Example: As an example, of 9,226 male GPs, the likelihood that TE was managed at any of the sample 100 encounters was 19.8% (95% CI: 19.0–20.6)

### Description of service characteristics

The vast majority of TE encounters were face-to-face consultations with the GP seeing the patient, and of these (S1 Table) the vast majority (80.9 per 100; 95%CI: 79.4–82.4) were claimable from the MBS/DVA (Federal Government Health Insurance schemes). The encounters were most commonly standard clinical consultations. There was a 9.6 (95%CI: 8.8–10.4) times greater likelihood that TE encounters would be covered by the worker’s compensation scheme (for work-related injury) than non-TE encounters (S2 Table).

There were 79.5 other health problems managed per 100 TE encounters and the most common are listed in Table 3. The most common concomitant problems were health conditions such as hypertension (4.8 per 100 TE encounters), depression (3.1), and lipid disorders (2.6). There were also a number of musculoskeletal conditions in the comorbidities managed at TE encounters, including bursitis/tendonitis/synovitis (not elsewhere classified) (2.1 per 100 TE encounters), osteoarthritis (2.0), shoulder syndrome (1.5), back complaint (1.5); sprain/strain (1.3), and carpal tunnel syndrome (1.0) (Table 3). Carpal tunnel syndrome and bursitis/tendonitis/synovitis (not otherwise specified) were co-managed during a TE encounter at a relative risk of 5.3 (95% CI: 3.7–7.5) and 2.0 (95% CI: 1.6–2.6) respectively compared with non-TE encounters (S2 Table).

**Table 3:** Most commonly managed problems other than TE^ at TE encounters (April 2000 –March 2015).

Problem label	n	per 100 encounters (95% CI)
Hypertension*	153	4.8 (4.1–5.6)
Depression*	100	3.1 (2.5–3.8)
Lipid disorders*	82	2.6 (2.0–3.1)
**Bursitis/tendonitis/synovitis NOS**	67	2.1 (1.6–2.6)
Osteoarthritis*	63	2.0 (1.5–2.5)
Prescription all*	54	1.7 (1.2–2.2)
Diabetes*	54	1.7 (1.2–2.2)
**Shoulder syndrome**	49	1.5 (1.1–2.0)
Back complaint*	48	1.5 (1.1–1.9)
Preventive immun/vacc/meds-all*	47	1.5 (1.1–1.9)
Gastro-oesophageal reflux disease*	44	1.4 (1.0–1.8)
Sprain/Strain*	42	1.3 (0.9–1.7)
**Upper respiratory infection, acute**	42	1.3 (0.9–1.7)
Female genital check-up*	37	1.2 (0.8–1.5)
Anxiety*	35	1.1 (0.7–1.5)
General check-up*	34	1.1 (0.7–1.4)
**Carpal tunnel syndrome**	32	1.0 (0.7–1.4)
Headache*	31	1.0 (0.6–1.3)
**All**	**2528**	**79.5 (76.1–82.8)**

NOS-not otherwise specified

^ ‘Problems other than tennis elbow’ whose rate was at least 1.0 per 100 tennis elbow encounters.

* indicates a grouping of multiple International Classification of Primary care rubrics, or of ICPC-2 Plus terms.[11]

### Management, including referrals to other health care providers and investigations

The majority of encounters for TE were directly managed by the GP (Table 4), either through procedural treatments (n = 1154; 36.3 per 100 TE problems managed) or provision of advice and education (n = 906; 28.5 per 100). Procedural treatments largely consisted of physical medicine/rehabilitation, and local injection/infiltration. Referrals to other health care providers (n = 432) were given at a rate of 13.6 per 100 TE problems, those to physiotherapy (9.3 per 100 TE problems managed) being the most common. Imaging tests were requested at a rate of 10.6 test orders per 100 TE problems while orders for pathology tests (e.g. full blood count, ESR, lipids, C reactive protein) were infrequent (3 per 100 TE problems).

**Table 4:** Treatments, referrals and tests ordered for tennis elbow (TE) problems (April 2000 –March 2015).

Category	Subgroup^	Number in each category or subgroup	Per 100 TE problems (95% CI)
**Total GP clinical treatments^^**	** **	**906**	**28.5 (26.6–30.3)**
			
**Total GP procedural treatments**	** **	**1154**	**36.3 (34.1–38.4)**
** **	Physical medicine/rehabilitation*	513	16.1 (14.6–17.6)
** **	Local injection/infiltration*	281	8.8 (7.7–9.9)
** **	Other therapeutic procedures/surgery NEC*	167	5.2 (4.1–6.4)
** **	Dressing/pressure/compression/tamponade*	116	3.6 (3.0–4.3)
** **	Repair/fixation-suture/cast/prosthetic device (apply/remove)*	70	2.2 (1.7–2.7)
** **	Other	7	—
**Total referrals to other health care providers^^^**	** **	**432**	**13.6 (12.3–14.8)**
** **	Physiotherapy	295	9.3 (8.3–10.3)
** **	Orthopaedic surgeon	48	1.5 (1.1–1.9)
** **	Rheumatologist	18	0.6 (0.3–0.8)
** **	Physician	11	0.3 (0.1–0.5)
** **	**Sports medicine practice**	9	0.3 (0.1–0.5)
** **	Occupational therapy	8	0.3 (0.1–0.4)
** **	Other	43	—
**Total pathology**	** **	**97**	**3.0 (1.9–4.2)**
** **	Full blood count	20	0.6 (0.4–0.9)
** **	C reactive protein	13	0.4 (0.2–0.6)
** **	Lipids	9	0.3 (0.1–0.5)
** **	ESR	8	0.3 (0.1–0.4)
** **	Other	47	—
**Total imaging**	** **	**338**	**10.6 (9.3–11.9)**
** **	Ultrasound;elbow	183	5.8 (4.9–6.6)
** **	X-ray;elbow	123	3.9 (3.2–4.6)
** **	Other	32	—

^ Only specifies those subgroups where the rate was at least 0.3 per 100 TE problems.

^^ Clinical treatments are primarily advice, education and counselling.

^^^ Of 3182 TE problems, 416 (13.1%) were each associated with 1 referral, and 8 (0.25%) with 2 referrals.

* indicates a grouping of multiple ICPC rubrics [11].

NEC: not elsewhere classified.

GP: general practitioner

ESR: erythrocyte sedimentation rate

There were 1903 medications prescribed/advised for over the counter purchase/supplied by the GP directly to the patient (Table 5) in the management of TE. Two thirds of these were oral and topical non-steroidal anti-inflammatories (NSAIDs) with oral NSAIDs accounting for approximately one half. Injected medications (mostly corticosteroids) were given at a rate of 14.1 per 100 TE problems and accounted for 23.6% of all TE medications. There was no evidence of a linear change in the rate at which injected medications were given for TE over the 15 years (S1 Fig).

**Table 5:** Medications^ provided, prescribed or recommended for tennis elbow (TE) problems at encounter (April 2000 –March 2015).

Medication group	Selected generic group^^	Number of medications	Per 100 TE problems (n = 3,182) (95% CI)	Percent of TE medications (n = 1,903) (95% CI)^^^
**All TE medications**		**1,903**	**59.8 (57.4–62.2)**	**100.0 (98.3–101.7)**
			
**All injected TE medications**		**449**	**14.1 (12.7–15.6)**	**23.6 (21.3–25.9)**
	Methylprednisolone	118	3.7	6.2
	Betamethasone systemic	107	3.4	5.6
	Triamcinolone	63	2.0	3.3
	Steroid injection nec	58	1.8	3.0
	Lignocaine	39	1.2	2.0
	Hydrocortisone systemic	35	1.1	1.8
	Local anaesthetic injection	17	0.5	0.9
**All topical TE medications**		**336**	**10.6 (9.4–11.7)**	**17.7 (15.9–19.4)**
	Diclofenac topical	250	7.9	13.1
	Piroxicam topical	28	0.9	1.5
**All oral or other TE medications**		**1,118**	**35.1 (33.2–37.1)**	**58.7 (56.2–61.3)**
	Diclofenac sodium systemic	235	7.4	12.3
	Celecoxib	138	4.3	7.3
	Meloxicam	138	4.3	7.3
	Ibuprofen	136	4.3	7.1
	Paracetamol	95	3.0	5.0
	Paracetamol/Codeine	58	1.8	3.0
	Rofecoxib	55	1.7	2.9
	Naproxen	53	1.7	2.8
	Diclofenac potassium	47	1.5	2.5
	Piroxicam oral	22	0.7	1.2
	NSAIDs	21	0.7	1.1

^ As classified in Coding Atlas of Pharmaceutical Substances (CAPS).

^^ Includes all CAPS generic groups whose rate was at least 0.5 per 100 TE problems; sum is less than subtotals of medication groups.

^^^ 1903 medications in total, associated with 1659 TE problems. The 95% CI analysis considers all 3182 problems, either with (n = 1659, 52.1%) or without (n = 1523, 47.9%) associated medication.

nec: not elsewhere classified.

## Discussion

This is the first survey of the rate of TE managed by GPs in Australia and shows that TE was managed at an estimated average of 242,000 encounters per year. To put this in context, this is similar to the number of GP encounters at which hip and knee osteoarthritis was managed in 2014[15]. From 2000 to 2015 there was a significant reduction in the proportion of GP encounters at which TE was managed. Possible explanations might include that: TE is not as common in the community; or, regardless of prevalence, TE is managed at a smaller proportion of an annually increasing number of GP encounters for other problems; or that more recently patients with TE are choosing to wait it out, self-treat or seek treatment elsewhere;[16]. It is tempting to speculate that clinical trials that have shown resolution over the long term in those who adopt a ‘wait and see approach’ have influenced the decisions of individuals who have TE, leading them to not bring it to the attention of their GP. Research to clarify this particular speculative relationship would help understand the observed trend.

The distributions of patient characteristics (e.g. age and sex) managed for TE are due in part to the distributions of these characteristics at all BEACH encounters [17]. Patients aged between 35 and 64 years constitute the major proportion of TE managed, and have the highest age-specific likelihoods of TE managed at GP encounters. This reflects the age groups frequently reported in clinical trials of TE [18]. In contrast to the age and sex characteristics reflecting those at all BEACH encounters, the TE encounters were almost 10 times as likely as non-TE encounters to be work related (as measured by workers compensation payment, S2 Table). This is not unexpected as work related factors feature strongly in this condition [1].

Most GPs who managed TE were male (69%, in part due to the preponderance of male GPs at all BEACH encounters, reflecting the sex distribution of the practising GP population)[17]. For male GPs, the GP sex-specific likelihood of managing TE at one or more of their 100 patient encounters was 20%, compared with 15% for female GPs (Table 2). This may in part be due to the higher percentage of female patients (who have a lower sex-specific likelihood of TE) managed by female GPs (compared with male GPs)[19].

GPs predominantly managed patients with TE by providing advice, education or counselling (29 per 100 TE problems) and procedural treatments (36 per 100 TE problems). While there are no Australian clinical guidelines against which to compare this practice, several clinical trials have reported that educating patients about the condition and self-management together with adopting a ‘wait and see’ approach will see resolution of TE over 6 to 12 months in the majority of patients [20, 21]. While this resolution takes some months to manifest, the ‘wait and see’ approach has been found to be superior to corticosteroid injections over the mid to long-term (i.e., 6–12 months), with fewer recurrences and a better recovery rate [6]. In the current study, referrals to other health care providers occurred at a rate of 14 per 100 TE problems, and these were mainly to physiotherapists. It seems that GPs prescribe a local injection or refer for physiotherapy at similar rates, but perform physical medicine/rehabilitation at a higher rate. There is evidence that physical therapies, typically performed by physiotherapists (exercise and mobilisation with movement) are superior to corticosteroid injections in terms of recovery and recurrence rates over the mid to long term [21].

It is interesting that the rate at which injections were given for TE over the 15 years did not change when the evidence over the past decade indicates that injections (particularly corticosteroid) are associated with delayed recovery and higher rates of recurrence [6, 18, 21, 22]. A better understanding of why injections remain used at a consistent rate could be the focus of research that might improve the translation of research findings into clinical practice.

It is widely accepted that tennis elbow is a diagnosis made on the basis of presenting signs and symptoms, with imaging reserved for cases in which there is a decision to exclude differential diagnoses such as injury of the radial collateral ligament and radio-humeral joint [23, 24]. Ultrasound was the most used imaging modality followed by plain X-ray, though at a rate of 10 per 100 TE cases it would appear that these are not used to make the diagnosis of TE. It is not possible to determine the GP’s reasons for the imaging from our data, but it is conceivable that the ultrasound might be used for soft tissue pathology about the elbow, including the common extensor tendon region or the radial collateral ligaments, and that X-ray might have been used to examine the bone and joint structures.

The concomitant management of musculoskeletal problems (e.g., bursitis, tendonitis, synovitis) of the shoulder, elbow and wrist (carpal tunnel syndrome), in some patients for whom TE was being managed is a finding consistent with other studies that highlight co-existing regional musculoskeletal conditions [2, 25]. This finding might inform a GP’s clinical reasoning in forming a prognosis, because it has been shown that TE patients who have concomitant neck and shoulder pain have a poorer prognosis [26]. Concomitant upper limb problems aligns with findings that TE patients have generally weaker muscles of the upper limb [27, 28] and suggest that optimal physical rehabilitation should entail a more general upper limb approach [29]. We did not have access to any of the further investigations and management for concomittant managed musculoskeletal conditons, which would be a recommendation for future interrogation of the Bettering the Evaluation of Care of Health program database.

We found that patients managed for TE at an encounter were also likely to be managed at that same encounter for other systemic health problems that commonly present to GPs [17] such as hypertension, lipid disorders and depression. This is consistent with evidence that shows that dyslipidaemia and adiposity are more frequently encountered in some tendinopathies [30] and tendinopathy presents in greater proportions of patients with obesity[31] and diabetes[32]. The clinical relevance of this finding pertains to the GP following guidelines that recommend exercise and physical activity in the management of systemic health problems[33, 34]. Prescribing the appropriate dose of exercise and physical activity is required, because injudicious dosing of physical activity and exercise is likely to provoke tendinopathy[35].

When drawing inferences from these results several constraints should be considered. First, the data collected for this study are limited to general medical practice in Australia and might not reflect encounters in other settings, nor how it is managed in other settings. Second, the data pertains to the conditions that were managed at a consultation and as such are not to be misconstrued as population prevalence of either the condition reported or other comorbidities. Third, the data are a snapshot in time of 100 consecutive consultations by about 1,000 different GPs in each year, for a period of 15 years and does not follow an individual patient over time, so it does not provide evidence of the condition's time course.

## Conclusion

TE is managed in Australian general medical practices at a rate that is commensurate with that for hip and knee osteoarthritis. As per general consensus the diagnosis made by the GP is essentially a clinical one without recourse to diagnostic tests or imaging. Assessment should pay particular attention to comorbidities such as other musculoskeletal problems or systemic illnesses as these will likely alter the overall management and prognosis of the patient. Management, consisting mainly of advice/education, some GP procedural treatments and referral to physiotherapy, largely follows what is recommended in the literature. That is, with the exception that there does not appear to be abatement of the use of corticosteroid injections in the 15-year census period, during which clinical trial research has not supported their use for TE. It appears that the time is right for the development of clinical guidelines for GPs.

## Supporting information

S1 Table Distribution of encounter types when tennis elbow (TE) managed at encounter (April 2000 – March 2015). (DOCX)

S2 Table For encounters where tennis elbow (TE) as managed (April 2000 – March 2015): the relative risk of worker’s compensation paid and other problems managed at encounter. (DOCX)

S1 Fig Likelihood (%) of at least 1 injection per tennis elbow problem (95% CIs). (DOCX)
