# Supplementary material for: No abatement of steroid injections for tennis elbow in Australian General Practice: A 15-year observational study with random general practitioner sampling
Source: PLoS One. 2017 Jul 20;12(7):e0181631. doi: 10.1371/journal.pone.0181631 (PMC5519163; doi:10.1371/journal.pone.0181631)
Supplement: S1 Table — (DOCX) [file pone.0181631.s001.docx]

**S1 Table: Distribution of encounter types when tennis elbow (TE) managed at encounter (April 2000 – March 2015)**

| **Encounter type** | Encounter subtype | Encounter subtype detail | Number* of encounters at which TE was managed | Rate per 100 encounters at which TE was managed (95% CI) |
| --- | --- | --- | --- | --- |
| **^Direct encounters** |  |  | 2962 | 99.4 (99.1–99.6) |
|  | No charge |  | 4 | 0.1 (0.0–0.3) |
|  | MBS/DVA items by GPs |  | 2412 | 80.9 (79.4–82.4) |
|  |  | Short surgery consultations | 5 | 0.2 (0.0–0.3) |
|  |  | Standard surgery consultations | 1880 | 63.1 (61.2–65.0) |
|  |  | Long surgery consultations | 351 | 11.8 (10.6–13.0) |
|  |  | Prolonged surgery consultations | 20 | 0.7 (0.4–1.0) |
|  |  | Home or institution visits (not RACF) | 12 | 0.4 (0.1–0.7) |
|  |  | Residential aged care facility (RACF) | 3 | 0.1 (0.0–0.2) |
|  |  | Chronic disease management items | 10 | 0.3 (0.1–0.5) |
|  |  | GP mental health care | 4 | 0.1 (0.0–0.3) |
|  |  | Health assessments | 3 | 0.1 (0.0–0.2) |
|  |  | Other items | 124 | 4.2 (3.2–5.1) |
|  | Worker's compensation |  | 533 | 17.9 (16.4–19.4) |
|  | Other paid (hospital, state etc) |  | 13 | 0.4 (0.2–0.7) |
| **Indirect encounters** |  |  | 19 | 0.6 (0.4–0.9) |
|  |  |  |  |  |

Notes:

*Missing data removed. Of 3181 encounters where TE was managed, the type of encounter was not recorded for 200.

^Direct encounters: where there is a face-to-face meeting of the patient and the GP

MBS: Medical Benefits Scheme

DVA: Department of Veteran Affairs
