# Supplementary material for: No abatement of steroid injections for tennis elbow in Australian General Practice: A 15-year observational study with random general practitioner sampling
Source: PLoS One. 2017 Jul 20;12(7):e0181631. doi: 10.1371/journal.pone.0181631 (PMC5519163; doi:10.1371/journal.pone.0181631)
Supplement: S2 Table — (DOCX) [file pone.0181631.s002.docx]

**S2 Table: For encounters where tennis elbow (TE) as managed (April 2000 – March 2015): the relative risk of worker’s compensation paid and other problems managed at encounter**

| **Characteristic (ICPC-2 code^3^)** | Relative risk of the characteristic occurring at a TE encounter vs at a nonTE encounter (95% CI) | Rao-Scott Chi-Square on 1 df | *P*-value |
| --- | --- | --- | --- |
| **Worker's compensation paid** | 9.6 (8.8–10.4) | 3671.7 | <0.0001 |
| **Carpal tunnel syndrome (N93)** | 5.3 (3.7–7.5) | 110.2 | <0.0001 |
| **Bursitis/ tendonitis/ synovitis Not Otherwise Specified (L87)** | 2.0 (1.6–2.6) | 35.6 | <0.0001 |

Note: TE was managed at 2.0% (1.8–2.2) of Worker’s Compensation encounters and at 0.18% (0.18–0.19) of non-Worker’s Compensation encounters
