# Supplementary material for: No abatement of steroid injections for tennis elbow in Australian General Practice: A 15-year observational study with random general practitioner sampling
Source: PLoS One. 2017 Jul 20;12(7):e0181631. doi: 10.1371/journal.pone.0181631 (PMC5519163; doi:10.1371/journal.pone.0181631)
Supplement: Supplementary file 3 [file pone.0181631.s003.docx]

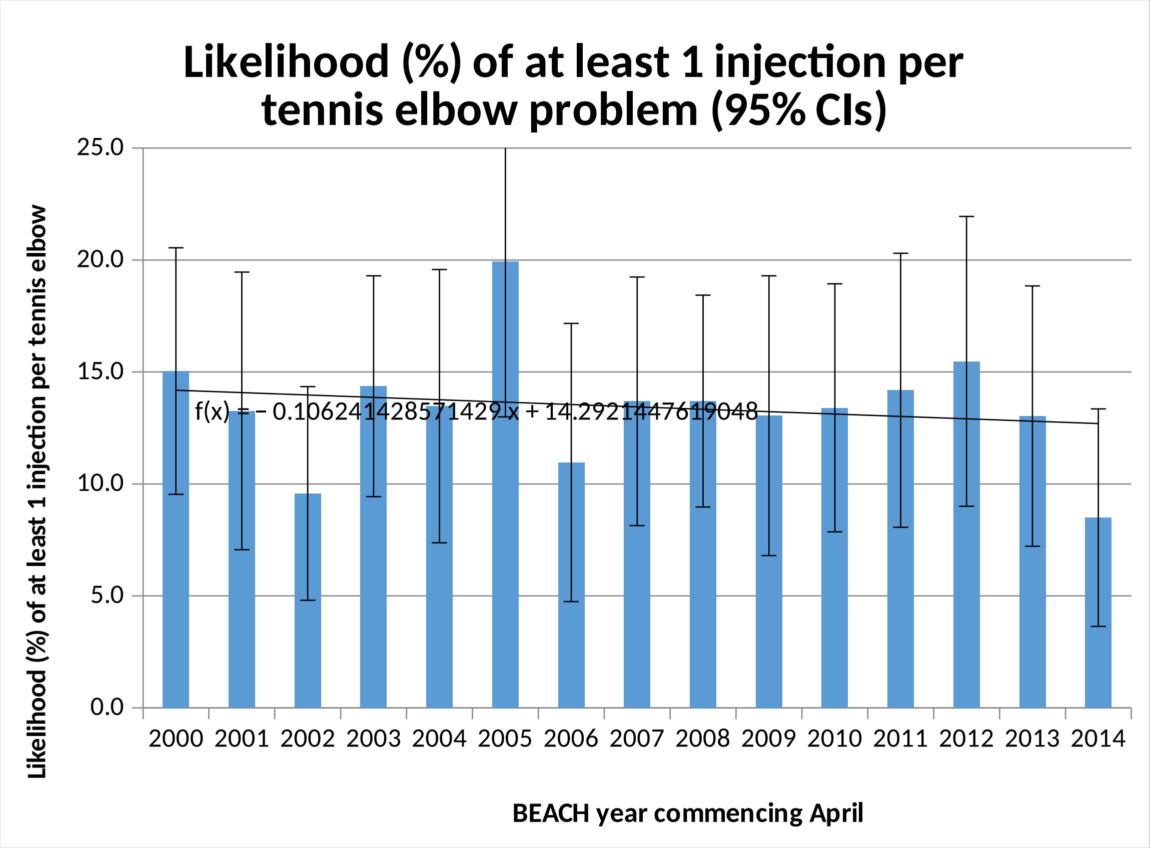
Likelihood (%) of at least 1 injection per tennis elbow problem (95% CIs)
25.0
20.0
15.0
10.0
5.0
0.0
Likelihood (%) of at least 1 injection per tennis elbow
f(x) = − 0.106241428571429 x + 14.2921447619048
2000 2001 2002 2003 2004 2005 2006 2007 2008 2009 2010 2011 2012 2013 2014
BEACH year commencing April
